# Cardiac Activation Maps Reconstruction: A Comparative Study Between Data-Driven and Physics-Based Methods

**DOI:** 10.3389/fphys.2021.686136

**Published:** 2021-08-26

**Authors:** Amel Karoui, Mostafa Bendahmane, Nejib Zemzemi

**Affiliations:** ^1^Institute of Mathematics, University of Bordeaux, Bordeaux, France; ^2^INRIA Bordeaux Sud-Ouest, Bordeaux, France; ^3^IHU-Liryc, Bordeaux, France

**Keywords:** data-driven approaches, physics-based approaches, ECGI inverse problem, cardiac activation mapping, neural networks, deep learning

## Abstract

One of the essential diagnostic tools of cardiac arrhythmia is activation mapping. Noninvasive current mapping procedures include electrocardiographic imaging. It allows reconstructing heart surface potentials from measured body surface potentials. Then, activation maps are generated using the heart surface potentials. Recently, a study suggests to deploy artificial neural networks to estimate activation maps directly from body surface potential measurements. Here we carry out a comparative study between the data-driven approach DirectMap and noninvasive classic technique based on reconstructed heart surface potentials using both Finite element method combined with L1-norm regularization (FEM-L1) and the spatial adaptation of Time-delay neural networks (SATDNN-AT). In this work, we assess the performance of the three approaches using a synthetic single paced-rhythm dataset generated on the atria surface. The results show that data-driven approach DirectMap quantitatively outperforms the two other methods. In fact, we observe an absolute activation time error and a correlation coefficient, respectively, equal to 7.20 *ms*, 93.2% using DirectMap, 14.60 *ms*, 76.2% using FEM-L1 and 13.58 *ms*, 79.6% using SATDNN-AT. In addition, results show that data-driven approaches (DirectMap and SATDNN-AT) are strongly robust against additive gaussian noise compared to FEM-L1.

## 1. Introduction

Cardiac activation mapping is an important tool for guiding medical treatments (catheter ablation) of different cardiac pathologies such as atrial fibrillation and ventricular tachycardia. It consists of inserting a catheter into the cardiac chambers and recording the electrical activity of the tissue at a given location. This process is repeated at multiple sites in order to cover a specific area or sometimes the whole cardiac chamber. Then, activation times are derived from these measurements by determining the point of maximum negative slope (IDT: intrinsic deflection time) or the point of maximal signal amplitude (Zipes and Jalife, 2009). The chosen technique depends on the signal nature: unipolar or bipolar. Finally, these activation times are interpolated to create a complete activation map of the heart chamber that helps the doctors localizing the target sites for catheter ablation.

This procedure is known to have some drawbacks. First, it doesn't allow to have a complete map of the chamber activation due to a reasonable limited number of stimulations. This raises different issues such as using inappropriate interpolation approach which can lead to irrelevant activation map reconstructions or mismapping catheter positions during the clinical procedure. Then, the most notable drawback is being invasive.

To address this problem, noninvasive electrocardiographic mapping suggests a battery of approaches to noninvasively reconstruct activation maps from noninvasively recorded body surface potentials (BSPs) and a heart-torso geometry reconstruction based on CT-Scan images using computational methods.

For example, in Cedilnik and Sermesant (2019), authors suggest a model personalization based on eikonal equation to compute activation times. In Van Dam et al. (2009), authors suggest to estimate activation times directly from BSPs using the equivalent double layer source model. In Yang et al. (2018), authors propose a novel formulation of ECGI inverse problem in the frequency domain. In other studies (Zemzemi et al., 2013; Giffard-Roisin et al., 2017), the kernel ridge regression is used to solve the inverse problem and reconstruct activation patterns. Besides, Duchateau *et al*. have suggested in Duchateau et al. (2016) to improve ECGI mapping by estimating activation delays between neighbor locations and construct an activation map from local activations and delay estimations. From another perspective, researches represented in Liu et al. (2006), Han et al. (2008), and Zhou et al. (2016) different approaches to reconstruct activation patterns using cardiac electric source imaging by identifying current densities in the heart.

However, these approaches use generally inverse methods that are known to be ill-posed and require applying regularization techniques on the solution. This yields smoothed solutions which makes it difficult to detect activation times.

Recent studies conducted a comparison between invasive and noninvasive mapping (Sapp et al., 2012; Cluitmans et al., 2017; Budanova et al., 2019; Duchateau et al., 2019). In Duchateau et al. (2019), authors provide a comparison between invasive contact mapping and noninvasive electrocardiographic imaging (ECGI) activation mapping using 59 clinically acquired activation maps. It states that ECGI mapping should be improved since the agreement between ECGI and invasive mapping results is poor. In fact, it shows that mean activation time error is 20.4 ± 8.6 *ms* and the between-map correlation is 3 ± 43%.

In this context, few researches were made in order to reach a better accuracy in localizing target sites for guiding catheter ablation using fewer invasive measurements Kania et al. (2018), Arrieula et al. (2019). Recent studies for localizing ventricular activation origin and ventricular tachycardia from the 12-lead ECG using machine learning methods (Zhou et al., 2019; Missel et al., 2020) have shown good performances in the identification of the arrhytmia origin. Godoy et al. suggested in Godoy et al. (2018a,b) a machine learning pipeline to localize atrial ectopic foci using the body surface potential integral maps (BSPMs). Another study developed a machine learning model to identify the site of origin of outflow tract ventricular arrhythmias from simulated patient-specific electrical information (BSPMs, ECGs,…) Doste et al. (2019). In Lozoya et al. (2019), authors suggest an image-based machine learning approach to detect cardiac radio-frequency ablation targets. In the same context, researchers conducted studies to improve efficacy of targeted persistant AF ablation (Alhusseini et al., 2019; Boyle et al., 2019). Recently, few reviews report all these studies and many others related to the application of machine learning approaches to arrhythmias and electrophysiology (Cantwell et al., 2019; Feeny et al., 2020; Trayanova et al., 2021).

In pursuit of the same goal, a previous study (Karoui et al., 2019a) suggests for the first time using artificial neural networks to estimate activation times directly from BSPs. It provides a proof-of-concept by building a model called DirectMap and assessing its performance using *in silico* data. Another recent study introduced a physics-informed neural networks for cardiac activation mapping (Sahli Costabal et al., 2020). In continuity with our previous works, we conduct a comparative study to evaluate quantitatively the performance of the data-driven methods: DirectMap (Karoui et al., 2019a) and the Spatial Adaptation of Time-Delay Neural Network (SATDNN-AT) (Karoui et al., 2019b) compared to the classic inverse method: Finite Element Method combined with L1-norm regularization (FEM-L1) (Karoui et al., 2018). The choice of these two methods is based on their performance results reported in Karoui et al. (2018) and Karoui et al. (2019b). The study is conducted using atrial paced *in-silico* data.

This manuscript is organized as follows: in section 2.1, we introduce the 3 methods, the synthetic data we use and the evaluation metrics. In section 3, we detail the results. Then, we end with a discussion, an evaluation of limitations and perspectives of this work, and we conclude in section 4.

## 2. Materials and Methods

### 2.1. Database

We build a synthetic paced-rhythm dataset of 101 simulations of BSPs and their correspondent activation time (AT) maps on the atrial outer surface. Each sample of BSPs and AT map corresponds to a single stimulation site randomly distributed on the atrial surface. We use the monodomain reaction-diffusion model to simulate the electrical wave propagation inside the heart. In order to simulate the BSPs, we first need to compute the extracellular potential in the heart (EGMs). Then, we use a Laplace's equation in the torso with a Dirichlet boundary condition on the heart-torso interface to compute the BSPs. For more details, see Zemzemi et al. (2013). Activation times are derived from the simulated EGMs by determining the IDT (Intrinsic Deflection Time) at each point of the atrial mesh. Let *u*_*i*_(*t*) be the unipolar signal at point *X*_*i*_ at time *t*, the IDT ${\hat{T}}_{i}$ is:

(1) $$\begin{array}{c} {\hat{T}}_{i}=arg\underset{t\in [0,T]}{\min}\frac{du_{i}(t)}{dt}, \end{array}$$

where *T* is the simulation duration. The finite element discretization of the realistic 3D atria-Torso geometry contains 264 nodes for the torso and 1994 nodes on the atrial surface. Each sample contains 400 time steps but the training is performed using only the first 200 time steps corresponding to the p-wave. The data used in the sections 3.1–3.3 are without additive noise.

### 2.2. Physics-Based Inverse Methods: FEM-L1

The study conducted in Karoui et al. (2018) evaluates the performance of fifteen algorithms combining different discretization and regularization techniques for reconstructing heart surface potentials (HSPs). According to this study, the finite element method combined with the L1-norm regularization (FEM-L1) of the current density over the heart surface provides the best results to solve the inverse problem of electrocardiography in terms of heart surface potential and pacing site localization. As it's mentioned in the state-of-the-art, the inverse problem is mathematically expressed as follows:

(2) $$\begin{array}{c} Ax=b \end{array}$$

where *A* is the transfer matrix generated using the finite element method, *b* is the boundary condition vector and *x* is the unknown potential vector.

Due to its ill-posedness, the inverse problem has to be solved using regularization. In this case, it turns out to minimize the objective function using L1-Norm regularization given by:

(3) $$\underset{x}{\min}\|Ax-b{\|}^{2}+{\text{λ}}^{2}\|\nabla x.n_{H}{\|}_{1},$$

where **n**_*H*_ is the outward unit normal to the epicardium surface and λ is the regularization parameter.

Using the Finite Element Method, we can define the Dirichlet-To-Neumann operator **D** satisfying:

(4) $$\left(\begin{array}{c} \frac{\partial x_{1}}{\partial n}\\ \vdots \\ \frac{\partial x_{n}}{\partial n} \end{array}\right)\begin{array}{c} =D\left(\begin{array}{c} x_{1}\\ \vdots \\ x_{n} \end{array}\right)\;, \end{array}$$

where **D** is an n-by-n matrix where *n* is the number of nodes in the heart surface.

Therefore, the objective function (3) can be expressed as follows:

(5) $$\underset{x}{\min}\|Ax-b{\|}^{2}+\lambda^{2}\|Dx{\|}_{1}.$$

Using an approximation of L1-Norm as an L2-norm, the linear problem to be solved is then simplified in a way that it can be seen as a first-order Tikhonov regularization.

In fact, following Karl (2005), we can smoothly approximate the L1-Norm of the derivative by:

(6) $$\begin{array}{c} \|Dx{\|}_{1}=\sum_{i=1}^{n}\left|{\left\lfloor Dx\right\rfloor }_{i}\right|\approx \sum_{i=1}^{n}\sqrt{|{\left\lfloor Dx\right\rfloor }_{i}{|}^{2}+\beta }, \end{array}$$

where *β* is a small constant satisfying *β* > 0 and ${\left\lfloor Dx\right\rfloor }_{i}$ the *i*^*th*^ component of the vector *Dx*.

This approximation leads to a set of equations whose resolution as *β* → 0 gives an estimate of the solution of (5) by solving the following linear problem:

(7) $$\begin{array}{c} \left[A^{T}A+\lambda^{2}D^{T}W_{\beta }(x)D\right]x=A^{T}b, \end{array}$$

where *W*_*β*_(*x*) is a diagonal matrix called **weight matrix**, expressed by:

(8) $$\begin{array}{c} W_{\beta }(x)=\frac{1}{2}diag\left[\frac{1}{\sqrt{|{\left\lfloor Dx\right\rfloor }_{i}{|}^{2}+\beta }}\right]. \end{array}$$

Then, thanks to the diagonality of *W*_β_(*x*), (7) can be written such that:

(9) $$\begin{array}{c} \left[A^{T}A+\lambda^{2}{\tilde{D}}^{T}(x)\tilde{D}(x)\right]x=A^{T}b, \end{array}$$

where $\tilde{D}\left(x\right)=\sqrt{W_{\beta }\left(x\right)}D$.

Computationally, the equation (9) is non-linear since the weighting matrix *W*_*β*_(*x*) depends on the solution *x*. To overcome this constraint, we suggest to use the Finite Element zero-order Tikhonov solution *x*_0_. Thus, we solve the problem expressed by:

(10) $$\begin{array}{l} \left[A^{T}A+\lambda^{2}{\tilde{D}}^{T}(x_{0})\tilde{D}(x_{0})\right]x=A^{T}b. \end{array}$$

### 2.3. Data-Driven Inverse Methods

In this section, we suggest two approaches for cardiac activation mapping based on artificial neural networks.

#### 2.3.1. Direct Cardiac Activation Mapping Using Electrocardiograms: DirectMap

**Figure 1:**
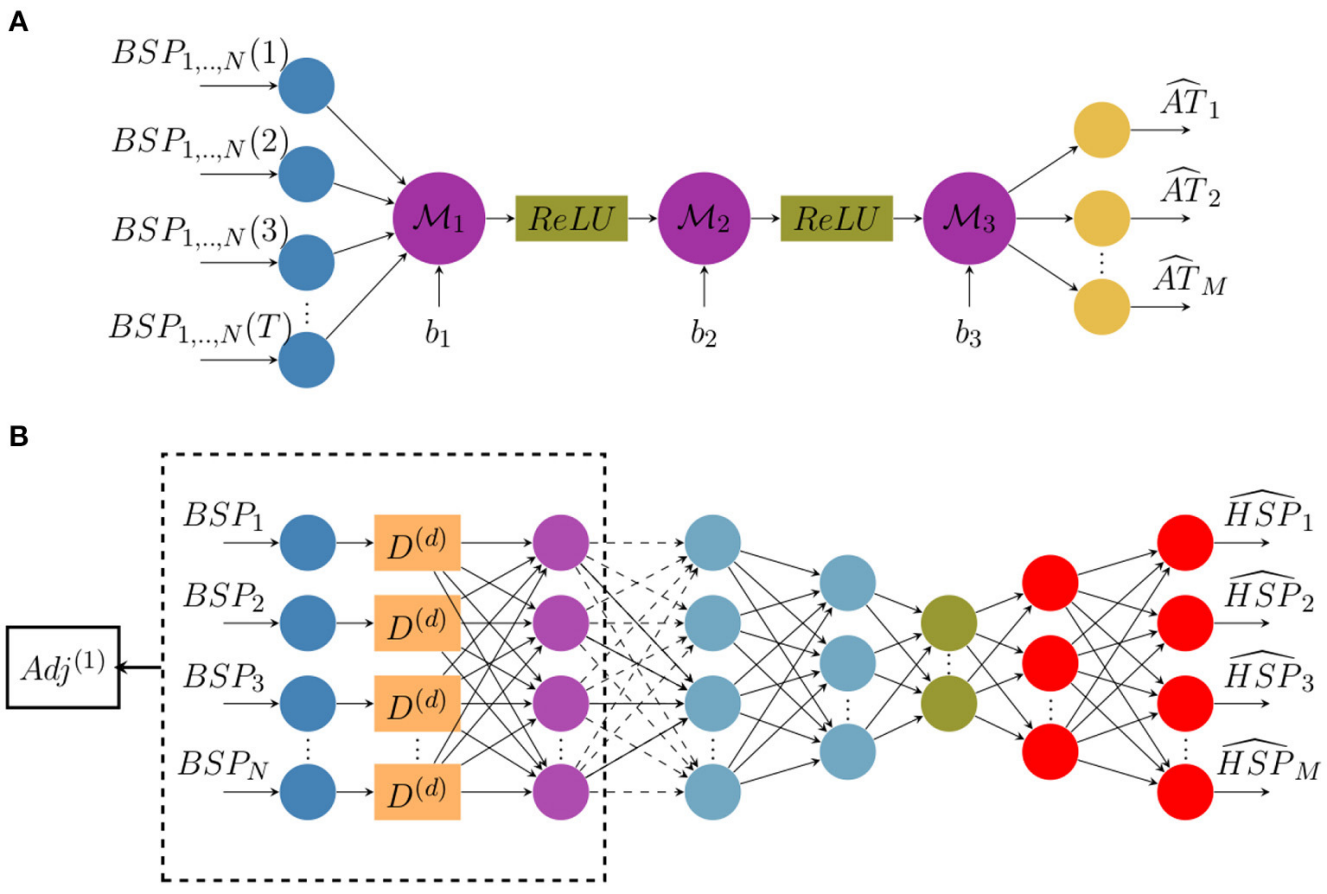
Architecture diagrams of **(A)** the direct activation mapping neural network (DirectMap) and **(B)** the spatial adaptation of the time-delay neural network (SATDNN-AT).

We suggest here to reconstruct activation time maps directly from ECGs without using electrograms (EGMs). To do so, we build a classic architecture of a neural network constituted of fully-connected and non-linear activation layers (ReLU). The network architecture is represented in Figure 1A where *N* is the number of measurement points on the body surface, *M* is the number of nodes on the heart surface and *T* is the sequence length.

To compute the optimal weights, the model has to minimize the following objective function with respect to the network parameters:

(11) $$\underset{{}_{{ℳ}_{1,2,3},b_{1,2,3}}}{\min}\|AT^{c}-AT^{e}{\|}^{2},$$

where *AT*^*c*^ is the estimated activation times vector and *AT*^*e*^ is the target one. According to the neural network architecture, *AT*^*c*^ is defined as follows:

(12) $$\begin{array}{l} AT^{c}=M_{3}\left[ReLU\left(M_{2}\left[ReLU\left(M_{1}BSP+b_{1}\right)\right]+b_{2}\right)\right]+b_{3}. \end{array}$$

Here, $M_{1,2,3}$ are network layer weights and *b*_1,2,3_ are biases.

The method consists of using the ECGs of a heartbeat sequence as input to the artificial neural network in order to estimate the corresponding activation time map.

A study is conducted over the neural network layers size. The aim is to determine the optimal model architecture for a given dataset. In order to guarantee a predefined accuracy on activation times reconstruction with the lower possible dataset size, we conducted a grid search procedure, allowing to determine simultaneously the maximal sparsity in the training data set and the optimal architecture of the neural network. We defined a threshold equal to 15 *ms* in terms of absolute error to choose the model with the minimal dataset size achieving a performance under this threshold. Results are reported in section 3.1.

#### 2.3.2. Cardiac Activation Mapping Using Reconstructed Electrograms by SATDNN-AT

The SATDNN-AT method was firstly introduced in Karoui et al. (2019b). It consists of reconstructing a heart surface potential at a time step *t* from body surface potential measurements at time step *t* and its previous values *t* − 1, *t* − 2, etc. The main idea is that the body surface potential at a time step t is highly dependent to its values at previous time steps *t* − 1, *t* − 2, etc. Thus, TDNN (Waibel et al., 1989) is a good candidate to get use of this dependence. In fact, each neuron in the TDNN uses the current and its *d* previous values of the BSP input to estimate the HSP target at the given time step *t* where *d* is the time-window size to fix.

Similarly to the temporal correlation, the heart surface potential in a given point P is strongly dependent on its recorded values at the adjacent points due to the propagation phenomenon. Hence, we use the spatial adjacency matrix as a representation of the relation between the target spatial location and its adjacent locations. According to Karoui et al. (2019b), this model called SATDNN-AT is made with two hidden layers. The first layer is identical to the TDNN where *D*^(*d*)^ is the time delay window of size *d* as represented in Figure 1B. Then, we perform an element-wise multiplication of the first layer output by the first order adjacency matrix *Adj*^(1)^. This allows, for each point, to only keep the weights corresponding to its adjacent points and reduces the others to zero.

In the interest of betterment, the model is here improved by building an autoencoder-like architecture represented in Figure 1B. It consists in building a bottleneck in the neural network that provides a compressed information representation which allows the model to ignore signal noise. The effect of this modification will be discussed in section 4.

### 2.4. Implementation

Data-driven models are implemented using Pytorch (Paszke et al., 2019). To train our models over labeled data, we use the mean squared error as an optimization criterion and the stochastic gradient descent as an optimization algorithm. K-fold cross validation (Refaeilzadeh et al., 2009) is used to evaluate the model performance on unseen data. It generally results on a less biased estimation of the target. The procedure consists on splitting the dataset on a training-validation dataset and a testing dataset. Then, K-fold cross validation is applied on the training-validation dataset. In fact, this latter is splitted into K groups. Each unique group is once kept as a validation dataset and all the remaining ones are used for training the model. In the end, the trained models are evaluated over the testing dataset. The training phase ends when the optimization criterion stops improving over the validation dataset. Hyper-parameters of the models are tuned empirically based on the performance of the models on the validation dataset. Learning rate and momentum are, respectively, 0.00001 and 0.8. The cross validation parameter K is equal to 4. Training and validation subsets are shuffled at each epoch of the training process. The time-window size *d* of SATDNN-AT is equal to 2.

Concerning the physics-based method, we developed the numerical tools into MUSIC software (Multi-modality Platform for Specific Imaging in Cardiology) (Cochet et al., 2014). More information about the MUSIC platform could be found in the following link: https://www.ihu-liryc.fr/en/music. MUSIC is intended for cardiac imaging processing, cardiac mapping analysis and electrocardiographic imaging inverse problem resolution.

For both potential based methods SATDNN-AT and FEM-L1, we post process the computed EGM signals using a Butterworth low-pass filter that eliminates the high frequency fluctuations.

### 2.5. Evaluation Criteria

To assess the precision of reconstructed activation maps, a point-based absolute activation time error (AATE) is computed as the absolute value of the difference between the exact and computed activation times at each point of the atrial mesh. Given $AT_{i,j}^{e}$ the exact activation time at point *j* of the simulation *i*, $AATE_{j}^{i}$ can be expressed as follows:

(13) $$\begin{array}{l} AATE_{j}^{i}=|AT_{i,j}^{e}-AT_{i,j}^{c}|, \end{array}$$

where $AT_{i,j}^{c}$ is the computed activation time at point *j* of the simulation *i*. Then, an average over all the mesh is computed. Pearson correlation coefficients (*CC*) are also computed between each activation time map pair for every simulation. To assess pacing site localization, we use the geodesic distance between estimated and exact pacing sites. These latter correspond, respectively to the node that has the minimum of estimated and exact activation times.

## 3. Results

### 3.1. Database Dependency Analysis

In this section, we present the results of the database dependency analysis performed on DirectMap. As it's mentioned in section 2.1, the database contains 101 simulations. To assess the database dependency, we suggest selecting subsets from the original dataset using the geodesic distance between stimulation sites as a selection criterion. In fact, we first compute the geodesic distances between all the stimulation sites corresponding to the 101 simulations. Then, we select the simulations whose distance between stimulation sites is above a fixed threshold. Using this approach, we succeed to select 9 subsets containing, respectively, 100, 85, 63, 50, 32, 25, 18, 16, 12, 10, and 8 simulations corresponding to a minimal distance between stimulation sites equal to 0.2, 1.2, 2.2, 3.2, 4.2, 5.2, 6.2, 7.2, 8.2, 9.2, and 10.2 mm, respectively. The subsets have been constructed by computing the minimal ($\underset{\text{dist}}{\min}$) and maximal ($\underset{\text{dist}}{\max}$) inter pacing sites distances, discretizing the interval $\left[\underset{\text{dist}}{\min},\underset{\text{dist}}{\max}\right]$ by 1*mm*, finding the subsets corresponding to each discretization step and removing the subsets containing <8 cases. This approach characterizes the spatial sparsity of each training data with its inter pacing site distance.

**Figure 2:**
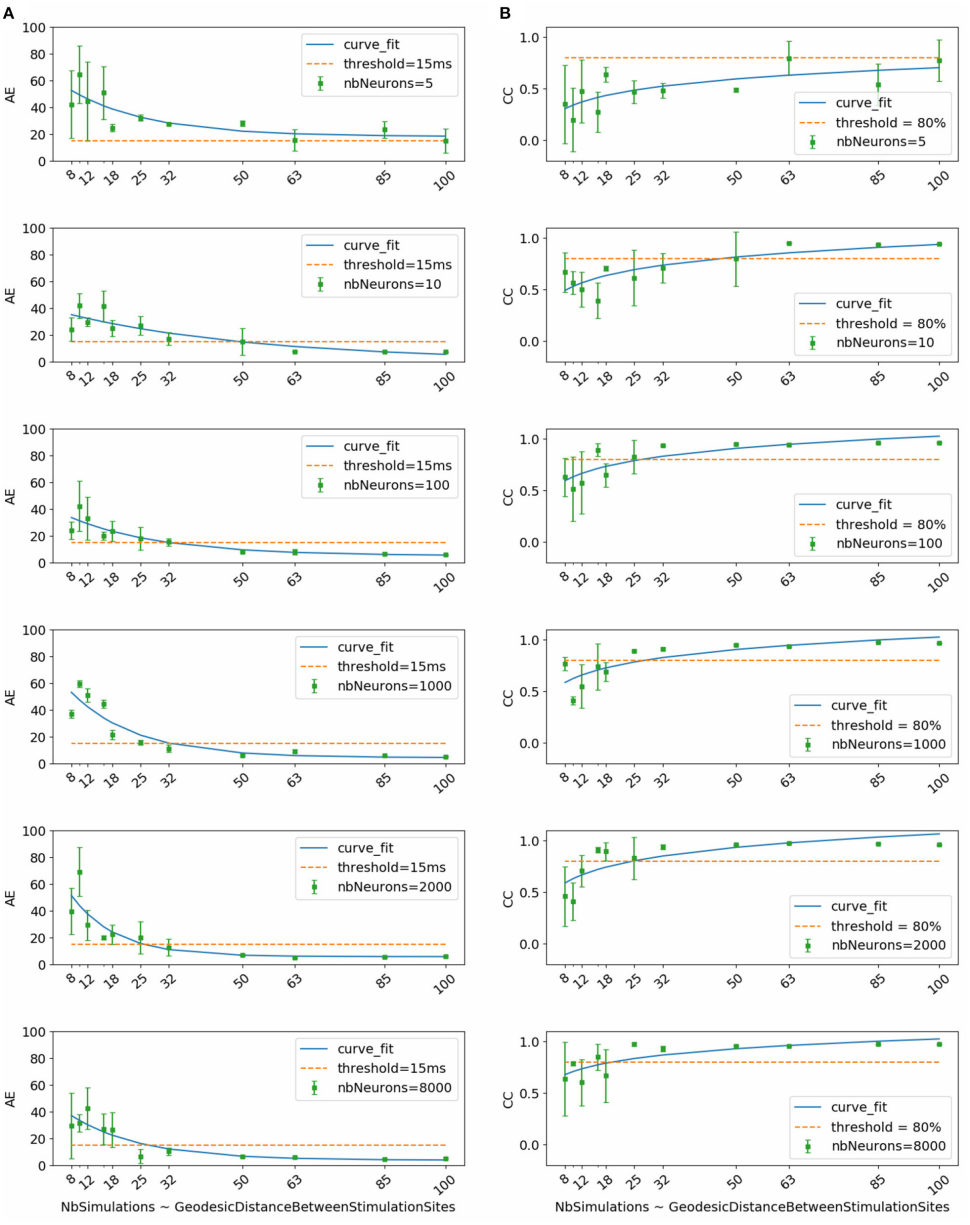
**(A)** Activation time absolute errors and **(B)** correlation coefficients with respect to dataset size. Each subfigure corresponds to trained neural network with the mentioned number of neurons per layer (nbNeurons).

Figure 2 shows the evolution of mean and standard deviation of absolute activation time error and correlation coefficient over the testing subset with respect to dataset size. Each row corresponds to the results obtained using, respectively (from top to bottom) 5, 10, 100, 1000, 2000, and 8000 neurons per hidden layer in the neural network.

To select the most appropriate model, we refer to the study conducted by Duchateau et al. (2019), where mean absolute error between invasive and noninvasive estimated activation times is equal to 20.04 *ms*. Considering the fact that this study is performed using *in-silico* data, we use a threshold in terms of absolute error equal to 15 *ms* represented in the Figure 2 by the dashed line. Another important selection criterion is the standard deviation. In fact, a high standard deviation means that results fluctuate between the folds and thus the model is not stable and vice versa.

By taking into account all these criteria, we observe that the model using 1000 neurons per hidden layer is the most stable for all the dataset sizes. We observe also that absolute activation time errors and correlation coefficients improve by increasing the dataset size. The sub-figure corresponding to the model using 1000 neurons per hidden layer shows that starting from 32 simulations, the results are below the threshold in terms of absolute error and above 80% in terms of correlation coefficient.

Therefore, results of the next phase of the study correspond to the chosen model using 1000 neurons per hidden layer and trained using the subset that contains 32 simulations. This subset corresponds to the case where the inter-pacing site distance is at least equal to 4.2 *mm*.

### 3.2. Cardiac Activation Mapping Results

**Figure 3:**
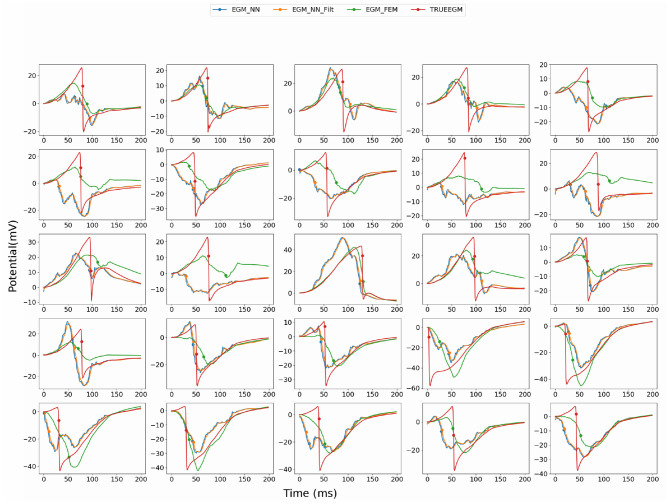
Comparison of the computed electrograms at different location of the atria: Exact solution (red line), solution using SATDNN-AT method (EGM_NN, blue line), SATDNN-AT with filter (EGM_NN_Filt, orange line) and FEM-L1 (EGM_FEM, green line). Points correspond to the estimated activation times. Each plot corresponds to a different node of the atria mesh.

In this section, we detail the results of the 3 methods and compare their performances based on the point-wise absolute activation time error and correlation coefficient. To do so, we choose the best model, in the sense of validation, from the 4 built models using the k-fold cross validation approach for DirectMap and SATDNN-AT. Figure 3 shows the absolute activation time error and correlation coefficient for every simulation of the training, validation and testing datasets using the methods DirectMap, SATDNN-AT and FEM-L1. If we concentrate on the testing results, we observe that DirectMap performs better than FEM-L1 and SATDNN-AT in terms of absolute errors and correlation coefficients. In fact, the average and standard deviation of the absolute errors and correlation coefficients are, respectively, equal to 7.20±3.42 *ms*, 93.2±2% using DirectMap, 14.60 ± 1.36 *ms*, 76.2 ± 5% using FEM-L1 and 13.58 ± 3.42 *ms*, 79.6 ± 11% using SATDNN-AT. These results are reported in Tables 1, 2.

**Table 1:** Means and standard deviations of absolute errors over training, validation, testing datasets and over all data (ms).

	**Training data**	**Validation data**	**Testing data**	**All data**
DirectMap	4.4 ± 3.1	3.9 ± 1.8	7.2 ± 3.4	4.9 ± 3.2
SATDNN-AT	8.4 ± 0.5	8.4 ± 0.5	15.1 ± 3.8	9.9 ± 3.3
FEM-L1	15.14 ± 1.9	15.47 ± 1.5	14.60 ± 1.3	15.08 ± 1.7
SATDNN-AT_Filt	6.6 ± 0.3	6.7 ± 0.5	13.58 ± 3.4	8.1 ± 3.2
FEM-L1_Filt	14.5 ± 1.8	14.8± 1.6	14.3 ± 1.3	14.4 ± 1.7

**Table 2:** Means and standard deviations of correlation coefficients over training, validation, testing datasets and over all data (%).

	**Training data**	**Validation data**	**Testing data**	**All data**
DirectMap	94.6 ± 3	94.9 ± 3	93.2 ± 2	94.3 ± 3
SATDNN-AT	95.8 ± 2	95.9 ± 2	79.6 ± 11	92.3 ± 8
FEM-L1	72.2 ± 11	73.2 ± 5	76.2 ± 5	73.2 ± 9
SATDNN-AT_Filt	96.9 ± 1	96.2 ± 1	83.1 ± 8	93.7 ± 7
FEM-L1_Filt	74± 10	73± 8	77.2 ± 4	74 ± 8

**Figure 4:**
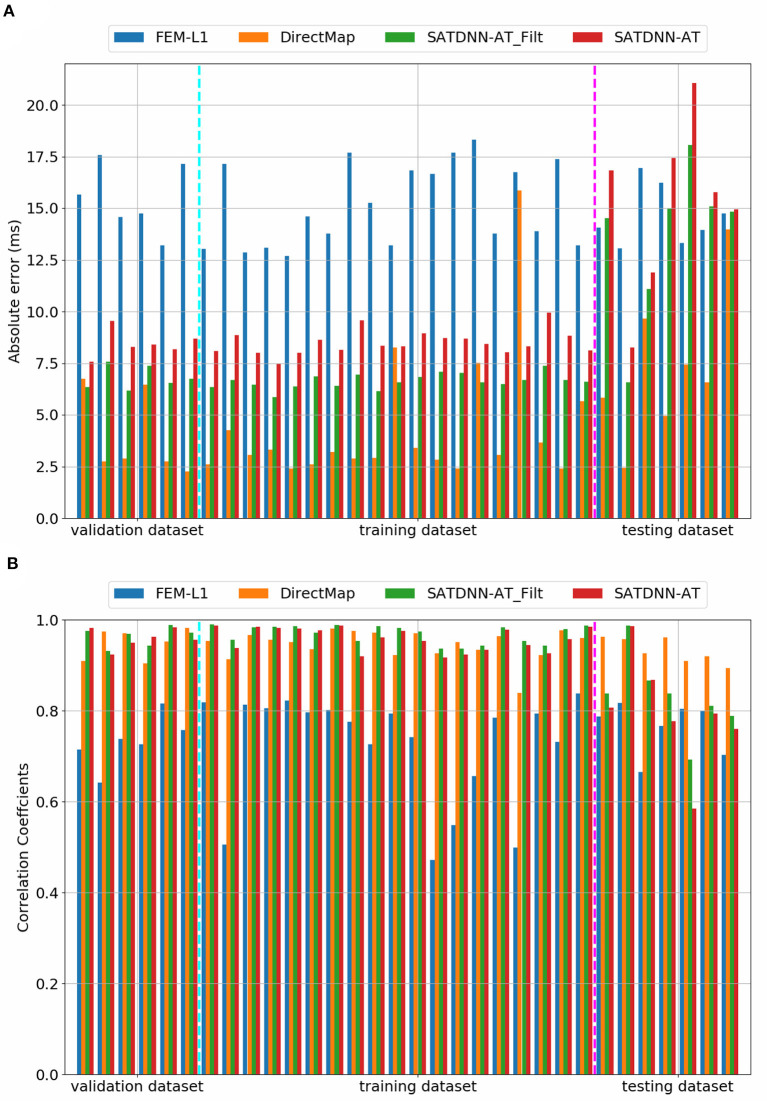
Comparison of **(A)** absolute errors and **(B)** correlation coefficients between exact and computed activation times using FEM-L1, DirectMap, SATDNN-AT and SATDNN-AT with filter for the 32 simulations.

When looking into SATDNN-AT results, we observe little fluctuations in the reconstructed EGMs which, for instance, can mislead the computation activation time estimation due to the fact that the AT is computed using the IDT. In order to solve this issue, we post process the computed EGM signals using a Butterworth low-pass filter that eliminates the high frequency fluctuations. Figure 4 represents exact and reconstructed EGMs using SATDNN-AT and SATDNN-AT after filtering at some selected points on the atrial surface. We observe that filtering either narrows or keeps the gap between exact and estimated activation times in almost all the nodes. In average, Tables 1, 2 show that results using the filtering technique are better than without filtering. To make a fair comparison, the same low-pass filter used for post-processing SATDDN-AT electrograms is applied to EGMs reconstructed by FEM-L1.

### 3.3. Pacing Site Localization Results

**Figure 5:**
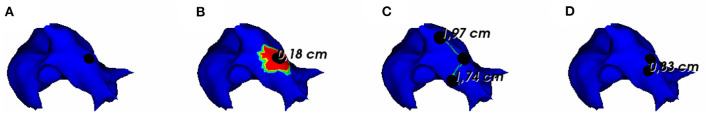
**(A)** Exact and reconstructed pacing sites using **(B)** FEM-L1, **(C)** SATDNN-AT with filter and **(D)** DirectMap for a test simulation. Numbers are geodesic distances between exact and estimated pacing sites.

To assess the pacing site localization performance, we use the geodesic distance between exact and reconstructed pacing sites as an evaluation metric. According to the observed results, there is an exception where we reconstruct the pacing site differently. In the case where the minimum value of activation times is shared by multiple nodes, as shown in Figure 5, we choose to take the gravity center of the nodes having the minimal AT value as the reconstructed pacing site.

**Figure 6:**
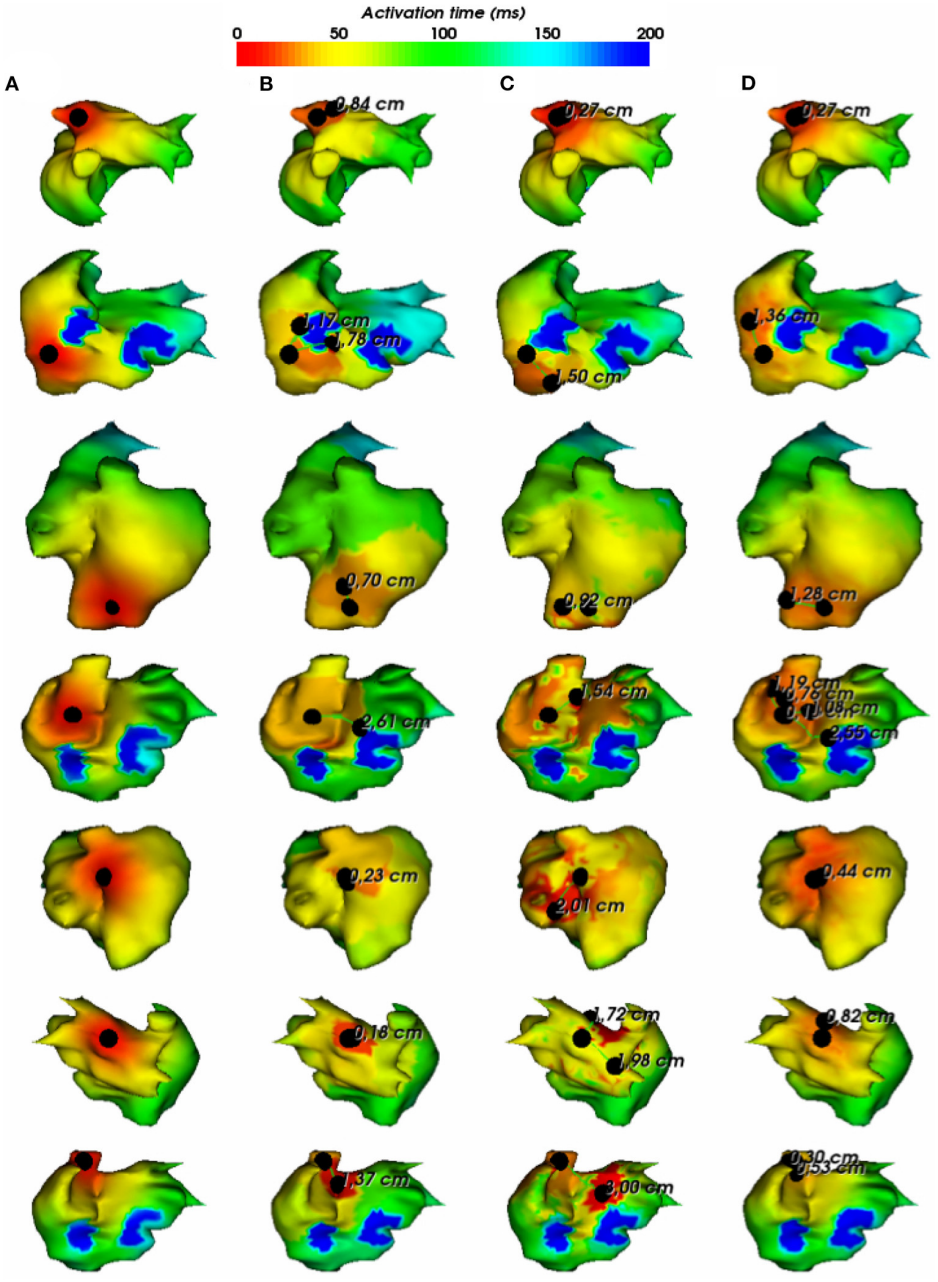
**(A)** Exact and estimated pacing sites using **(B)** FEM-L1, **(C)** SATDNN-AT with filter and **(D)** DirectMap for 7 different test simulations. Numbers are geodesic distances between exact and estimated pacing sites.

In Figure 6, we show the simulations of the testing dataset with the reconstructed pacing sites and the geodesic errors. We observe that in average, geodesic distances using FEM-L1, SATDNN-AT and DirectMap are, respectively, 9.5 *mm* ± 8.1, 13.2 *mm* ± 5.7, and 7.6 *mm* ± 4.2. We conclude that DirectMap outperforms the two other methods in terms of pacing site localization.

### 3.4. Robustness Against Added Gaussian Noise to the Testing Data

To assess and compare the robustness of the three methods against additive Gaussian noise, we represent in this section their results in terms of absolute activation time errors and correlation coefficients after adding to ECGs different noise levels in the range between 5 and 50% of the maximum signal amplitude. These tests are performed only on the testing data. Tables 3, 4 show that DirectMap is insensitive to noise addition in terms of both absolute error and correlation coefficient. Besides, SATDNN-AT is more robust than FEM-L1 against additive noise.

**Table 3:** Means and standard deviations of absolute activation time errors of the testing dataset with respect to noise level (ms).

**Noise (%)**	**0**	**5**	**10**	**30**	**50**
DirectMap	7.2 ± 3.4	7.2 ± 3.4	7.2 ± 3.4	7.2 ±3.3	7.2 ± 3.3
SATDNN-AT	15.1 ± 3.8	15.6 ± 3.5	16.4 ± 5	19.1 ± 3.6	21.8 ± 5
FEM-L1	14.6 ± 1.3	39.4 ± 4.4	43.3 ± 4	48 ± 3	49.5 ± 2.1
SATDNN-AT_Filt	13.5 ± 3.4	13.7 ± 3.4	13.8 ± 3.3	15.1 ± 3.1	16.3 ± 3.6
FEM-L1_Filt	14.6 ± 1.3	26.9 ±3.6	36.4 ± 3.1	45.5 ± 2.5	46.4 ± 3.5

**Table 4:** Means and standard deviations of correlation coefficients of the testing dataset with respect to noise level (%).

**Noise (%)**	**0**	**5**	**10**	**30**	**50**
DirectMap	93.2 ± 2	93.2 ± 2	93.2 ± 2	93.2 ± 2	93.2 ± 2
SATDNN-AT	79.6 ±11	79.6 ± 6	75.5 ± 7	72± 8	64.6± 14
FEM-L1	76.2 ± 5	23.9 ± 6	11.2 ±3	3.2 ± 6	-1.5 ± 9
SATDNN-AT_Filt	83.1 ± 8	82.3 ± 8	81.7 ± 8	75.9 ± 9	70.5 ± 13
FEM-L1_Filt	76.2 ± 5	49.5 ± 6.3	26.2 ± 7	11.2 ± 3.1	7.5 ± 6.1

**Figure 7:**
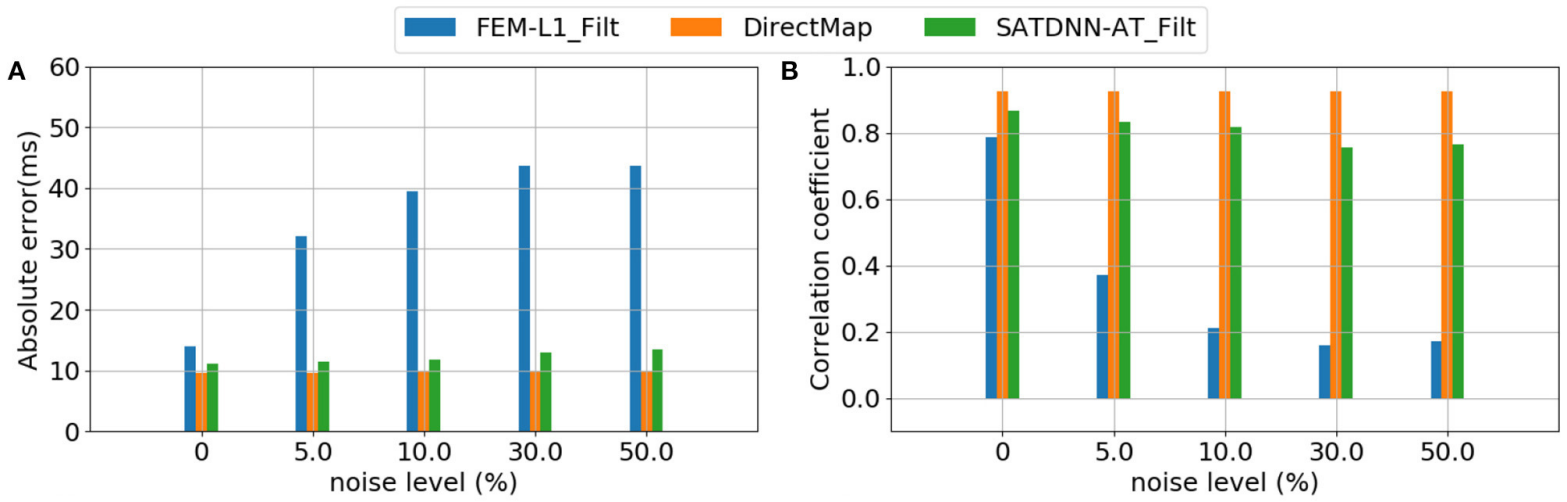
Evolution of **(A)** absolute errors and **(B)** correlation coefficients between exact and estimated activation times with respect to noise level using FEM-L1, DirectMap and SATDNN-AT.

Considering that the 3 methods behave the same way for all the simulations, Figure 7 represents results of a simulation of the testing dataset that confirms the previous deductions. In fact, we observe that FEM-L1 absolute error deteriorates from 15 to 32 *ms* then from 32 to 43*ms* for, respectively, 5 and 50% of noise level. The same applies to correlation coefficient that decreases from 79 to 38% then from 38 to 18% for, respectively, 5 and 50% of noise.

**Figure 8:**
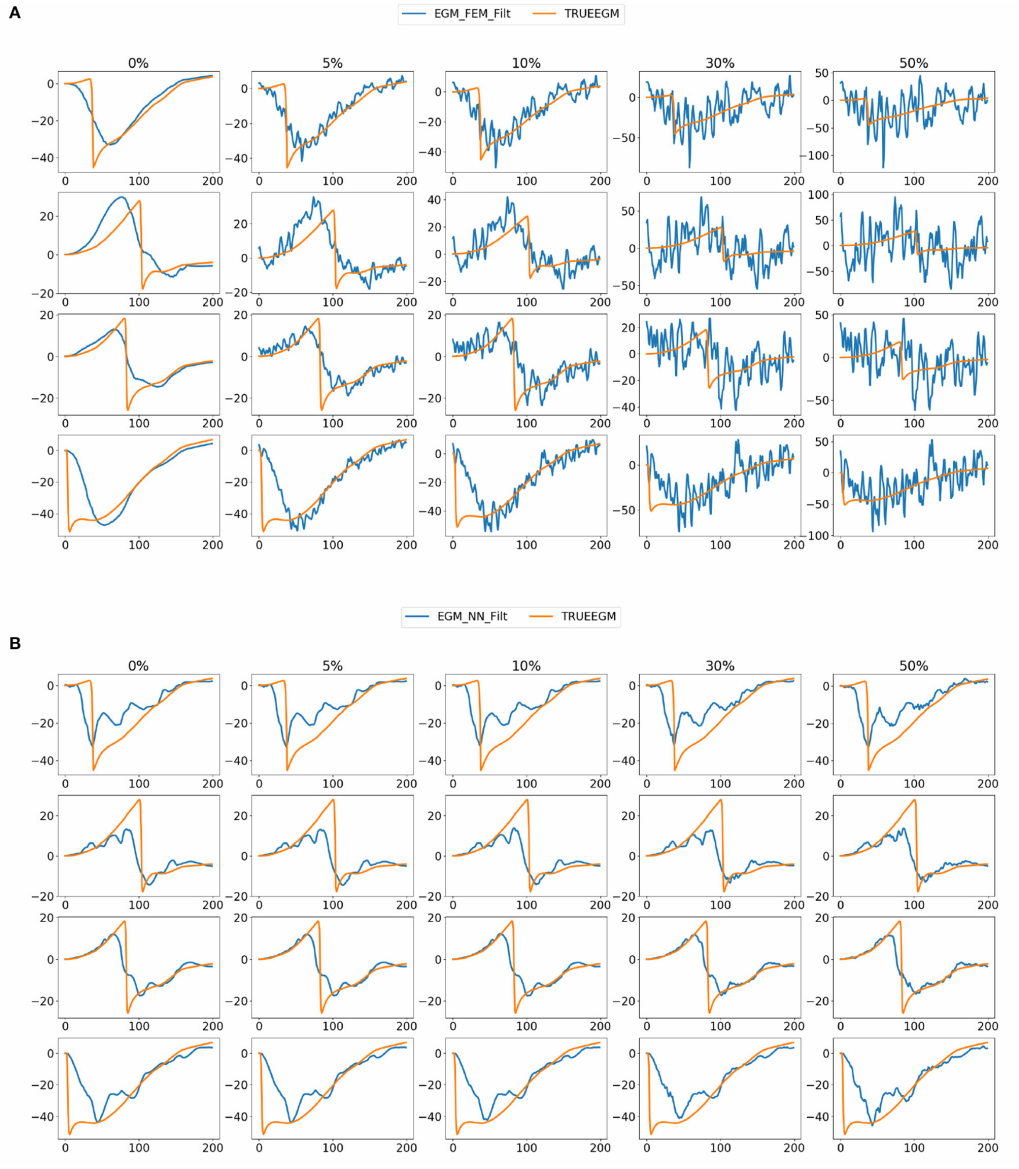
Exact and estimated electrograms using **(A)** FEM-L1 (EGM_FEM_Filt) and **(B)** SATDNN-AT (EGM_NN_Filt) with respect to the added noise level.

Figure 8 shows exact and estimated electrograms by FEM-L1 and SATDNN-AT using different noise levels going from 5 to 50%. We observe that the reconstruction quality of the electrograms using FEM-L1 deteriorates proportionally to the added noise level. However, the reconstructed electrograms using SATDNN-AT are slightly affected by the added Gaussian noise, which explains the difference between SATDNN-AT and FEM-L1 results in terms of activation time estimation.

### 3.5. Robustness Against Added Gaussian Noise to the Training Data

**Figure 9:**
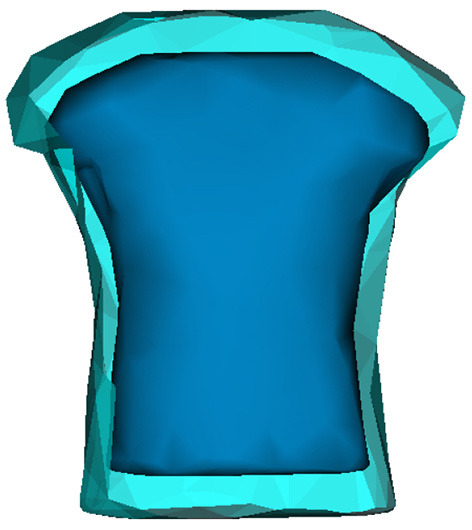
Inflated torso geometry generated by inflating the original geometry by a factor 1.2.

In this section, we study DirectMap model performance when trained and tested using noisy data. To do so, we generate noisy ECGs by adding 25% of noise. Then, activation maps are contaminated by adding a uniformly distributed noise between −5 and 5 *ms*, −10 and 10 *ms*, −20 and 20 *ms*, −30 and 30 *ms*. Figure 9 presents the average performance of the trained models using the noisy data with respect to the intensity of the added noise. We observe that the model performance deteriorate when the noise intensity increases. The mean absolute activation time error increases from 8.8 to 19.02*ms* and the correlation coefficients decreases from 96 to 80% when using ±5 and ±30 *ms* of noise, respectively.

### 3.6. Robustness Against Geometric Uncertainties During the Testing Phase

**Figure 10:**
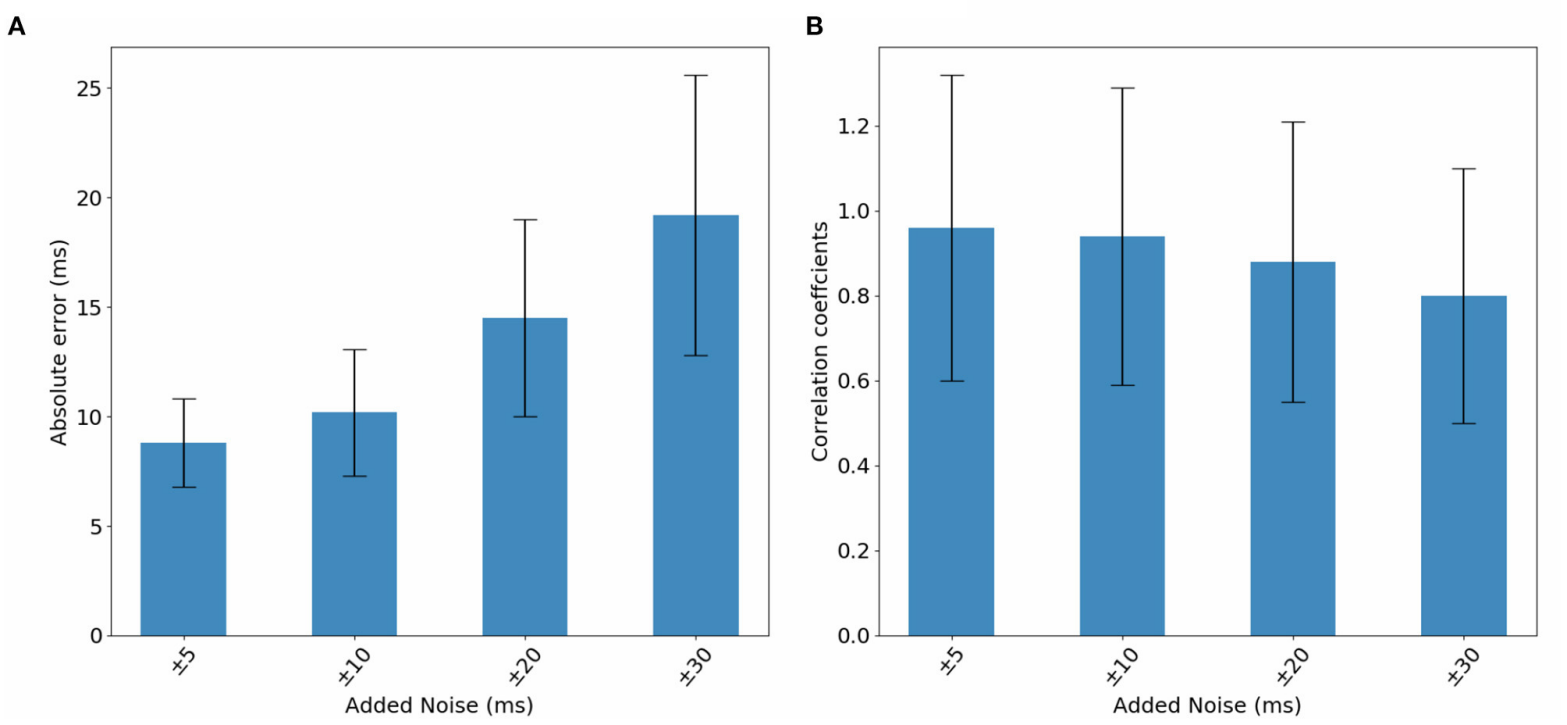
Bar graphs of the evolution of **(A)** absolute errors and **(B)** correlation coefficients with respect to noise added to activation maps. The results correspond to the testing phase.

To assess the robustness of the methods against geometric uncertainties, we modify the torso geometry by applying an inflation of a 1.2 factor as represented in the Figure 10. ECGs are simulated by solving the forward problem using the inflated geometry.

First, the modified ECGs are used to test the initial model DirectMap. Mean absolute error and correlation coefficient are equal to 14.08 ± 2.38 *ms* and 94.2 ± 35%, respectively. Using FEM-L1, results are 25.9 ± 5.6 *ms* and 43.7 ± 20.1%. Finally, we observe an absolute activation time error and a correlation coefficient equal to 23.5 ± 6.2 *ms* and 57.8 ± 11.9% using SATDNN-AT. To plot a complete comparison between the methods, we compute the evaluation metrics for SATDNN-AT and FEM-L1 after filtering. Results are 24.3 ± 5.2 *ms* and 53.5 ± 22.1% using FEM-L1 with filter and 21.2±4.7*ms* and 66.8±7.8% using SATDNN-AT with filter.

Then, Table 5 reports the evolution of absolute errors and correlation coefficients with respect to noise added to activation maps using the inflated torso geometry during the testing phase. We observe a deterioration in terms of absolute errors and correlation coefficients. The absolute error increases when using a noise between −5 and 5 *ms* from 17.6 *ms* to 19.9*ms* when we add a noise between −30 and 30 *ms*. The correlation coefficient decreases from 91.5 to 85.9%.

**Table 5:** Means and standard deviations of absolute errors and correlation coefficients of the inflated testing dataset with respect to noise added to activation maps.

**Noise (ms)**	**5**	**10**	**20**	**30**
AATE (ms)	17.6 ± 2.6	17.7 ± 2.7	18.6 ± 3.1	19.9 ± 3.8
CC (%)	91.5 ± 33	91 ± 33	88.9 ± 33	85.9 ± 31

## 4. Discussion and Conclusion

This study addresses two different issues: First, it studies the DirectMap generalization performance with respect to dataset size and the neural network architecture. Then, it compares DirectMap with two methods of the state-of-the-art cardiac activation mapping. The results confirm that the larger the dataset, the greater the performance. According to Duchateau et al. (2019), mean activation time absolute error using non-invasive cardiac activation mapping methods assessed on clinical data is equal to 20.04 *ms*. So, by fixing a threshold equal to 15 *ms* we deduce that using 32 simulations as a training dataset provides a great generalization performance.

Based on these results, a comparison study is conducted between DirectMap, SATDNN-AT and FEM-L1. It shows that DirectMap outperforms the two other methods. In terms of cardiac activation mapping, DirectMap achieves an improvement of nearly 7 *ms* in absolute error and, respectively, 10%, 17% in terms of correlation coefficient compared to SATDNN-AT and FEM-L1. A robustness analysis against noise was also conducted. First, it shows that DirectMap is strongly robust against eventual additive gaussian noise present in ECGs compared to SATDNN-AT and FEM-L1. Results show also that SATDNN-AT is more robust than FEM-L1 whose performance massively deteriorates. This study shows that data-driven methods are more robust than physics-based methods. This is due to the use of auto-encoder architecture which is known for its great performance in denoising data. In fact, it allows the neural network to learn from a reduced representation of the input information by ignoring noise features. Second, DirectMap performance was assessed when trained and tested using noisy data. As expected, the study shows that the performance deteriorates proportionally to the added amount of noise but it is still under the fixed threshold even when the noise added to activation maps ranges between −20 and 20 *ms*. Geometric uncertainties were also considered by inflating the torso geometry by a 1.2 factor. Testing the different approaches with these data shows a decline in the evaluation metrics. Nevertheless, DirectMap still achieves the best performance compared to FEM-L1 and SATDNN-AT.

Although DirectMap has promising results compared to SATDNN-AT and FEM-L1, many limitations are still to be addressed in future works. First, we have to admit that the built model has a basic neural network architecture which can be improved to meet the complexity of the problem. We have to notice that the size of the database has been optimized on the basis of DirectMap performance results and used later to evaluate the performance of the two other methods. This doesn't affect the FEM-L1 results. However, this choice might not be optimal for SATDNN-AT. Then, as we mentioned in Karoui et al. (2019a), tests are performed using perfect data with the same heart-torso geometry which is not compatible with real cases. So, geometry standardization would be one step forward in data-driven cardiac activation mapping. We also have to notice that using intrinsic deflection time as the computation method of activation time from the inverse solution computed with SATDNN-AT and FEM-L1 may not be optimal to compare these two methods to DirectMap. Besides, we observe that low-pass filtering of the inverse solutions EGMs improved the reconstruction of the activation maps. Moreover, since cardiac activation mapping is a diagnostic tool of cardiac diseases, our model would be more credible if trained and tested using data illustrating some specific cardiac pathologies. Like all the methods used in ECGI mapping, in order to take into account real-life data acquisition inaccuracies, it's important to quantify the performance of the model with respect to uncertainties such as misplacement of electrodes, shift and/or rotation of the atrial geometry within the body volume, different forward and inverse calculations, different electrode setups and number of electrodes, for example using standard 12-lead ECG instead of BSPMs. Geneser et al. (2007), Fikal et al. (2019), Tate et al. (2021), and Multerer and Pezzuto (2021) Finally, our study is still a proof-of-concept until sufficient clinical data would be available to validate our results.

Even though our model achieves valuable results, it is still not applicable in clinical cases due to the high number of required stimulations. To address this issue, future works will focus on data augmentation techniques in order to enrich the dataset without performing many pacings. One of the options is to combine data-driven and physics-based methods as it's presented in a recent study conducted by Sahli Costabal et al. (2020). After proving the feasibility and applicability of DirectMap, this work attests that it outperforms at least two of state-of-the-art methods: SATDNN-AT and FEM-L1. In summary, this study is encouraging and suggests that DirectMap technique needs further investigation and may have potential to become a useful noninvasive cardiac activation mapping tool.

## Data Availability Statement

The data analyzed in this study is subject to the following licenses/restrictions: The only restriction is related to the size of the data base. Requests to access these datasets should be directed to Nejib Zemzemi, nejib.zemzemi@inria.fr.

## Author Contributions

NZ, AK, and MB designed the study and wrote the manuscript. AK and NZ conducted the numerical simulation. All authors contributed to the article and approved the submitted version.

## Conflict of Interest

The authors declare that the research was conducted in the absence of any commercial or financial relationships that could be construed as a potential conflict of interest.

## Publisher's Note

All claims expressed in this article are solely those of the authors and do not necessarily represent those of their affiliated organizations, or those of the publisher, the editors and the reviewers. Any product that may be evaluated in this article, or claim that may be made by its manufacturer, is not guaranteed or endorsed by the publisher.
